# The prevalence of transmitted HIV drug resistance among MSM in Anhui province, China

**DOI:** 10.1186/1742-6405-11-19

**Published:** 2014-07-08

**Authors:** Yuelan Shen, Bin Su, Jianjun Wu, Yizu Qin, Lin Jin, Lifeng Miao, Aiwen Liu, Xiaoli Cheng

**Affiliations:** 1 Department of HIV&AIDS, Anhui Provincial Center for Disease Control and Prevention, Hefei 230601, Anhui, China

**Keywords:** HIV, Prevalence, Transmitted drug resistance, Treatment-naive, MSM

## Abstract

**Background:**

To optimize treatment regimens, we assessed human immunodeficiency virus (HIV) diversity and the prevalence of transmitted drug resistance (TDR) among men who have sex with men (MSM) in Anhui province, China.

**Methods:**

A total of 139 MSM who were newly diagnosed and antiretroviral treatment-naive were enrolled in Anhui in 2011. A partial *pol* fragment was amplified and sequenced, and HIV subtypes were determined by phylogenetic analyses. Surveillance/transmitted drug resistance mutations (SDRMs) were identified according to the 2009 World Health Organization (WHO) list.

**Results:**

A total of 133 (95.7%) samples were successfully amplified and sequenced. Based on phylogenetic analyses of the *pol* fragment, CRF01_AE accounted for 55.6% (74/133) of the infections, followed by CRF07_BC with 32.3% (43/133), B with 5.3% (7/133), and unique recombinant forms with 6.8% (9/133). A total of 3.0% (4/133) of the subjects were found to harbor HIV variants with SDRMs, including 1.5% with NRTI-related mutations and 1.5% with NNRTI-related mutations. PI-related mutations were absent. The SDRMs included L210W (1.5%), Y181C (0.8%), and G190A (0.8%).

**Conclusions:**

In Anhui, CRF01_AE strains contributed to most of the HIV infections among MSM, and the prevalence of TDR was relatively low in this population. Further studies should be performed to evaluate the trend of TDR among MSM in Anhui and to inform first-line antiretroviral treatment.

## Introduction

Globally, 34.0 million people were living with HIV by the end of 2011. An estimated 0.8% of adults 15–49 years of age worldwide are currently living with HIV-1 [1]. Studies suggest an increasing HIV-1 prevalence among men who have sex with men (MSM), and some evidence indicates that the global prevalence of HIV-1 infection among MSM has increased from 2010 to 2012 [2,3]. The HIV-1 epidemic in China has also increased. By the end of 2011, there were 780,000 people in China infected with HIV-1, with a national prevalence rate of 0.058%. Of the infected individuals, 63.9% were infected through sexual transmission, including 46.5% through heterosexual transmission and 17.4% through homosexual transmission [4]. Among the estimated 48,000 newly diagnosed cases in 2011, heterosexual transmission accounted for 52.2%, homosexual transmission for 29.4%, and injection drug users (IDUs) for 18.0% of the cases [4].

Anhui province, located in south-eastern China, neighbors Henan province and was defined as an HIV-1 epidemic focus where most infections were caused by former commercial blood donation [5]. However, newly diagnosed infections have rapidly increased through homosexual transmission among MSM in recent years [4]. To date, 768 MSM have been reported to be infected with HIV in Anhui province.

According to the national guidelines for antiretroviral (ARV) treatment in China, HIV-1-infected persons should receive antiretroviral therapy (ART) when their CD4 counts are less than 350 cells/μL. Extensive use of ARV drugs has led to the increasing transmission of HIV variants with drug resistance mutations that can be detected in individuals before the initiation of treatment [6-9], and gives rise to a reduction in efficacy of first-line ART [10]. From the beginning of national scale-up of combined antiretroviral therapy in China, many HIV drug resistance surveys have been conducted, and viral mutations conferring resistance to protease inhibitors (PIs) and/or reverse transcriptase inhibitors (RTIs) has been found in both ARV-treated patients and treatment-naive patients [11-15]. However, little is known about mutations associated with HIV-1 resistance to PIs and/or RTIs in newly diagnosed ART-naive MSM in Anhui. To provide information about the epidemic trends of HIV and to optimize the treatment strategies in Anhui, the prevalence of surveillance/transmitted drug resistance mutations (SDRMs) was evaluated in ART-naive MSM who were newly diagnosed as HIV-infected in 2011.

## Materials and Methods

### Study population and specimens

A cross-sectional study was conducted among newly diagnosed HIV-infected MSM in 15 prefectures of Anhui in 2011, according to guidelines of the Anhui Center for Disease Control and Prevention (CDC), which provides HIV/AIDS treatment and prevention services through education, quality services, and compassionate care to those with HIV/AIDS. Our research included the following 15 cities: Fuyang, Bozhou, Huaibei, Huainan, Suzhou, Bengbu, Hefei, Liu’an, Anqing, Huangshan, Xuancheng, Ma’anshan, Wuhu, Tongling and Chizhou. All newly diagnosed MSM with HIV were enrolled, except for MSM with known exposure to ARV drugs. All subjects were included anonymously and provided written informed consent. Demographic data were collected through direct interview using a standardized questionnaire prior to sample donation. Blood samples were collected from 139 individuals. Plasma was isolated within 6 hours of sampling and stored at −80°C until HIV drug resistance genotyping. The study was reviewed and approved by the local Ethical Review Committee of the Anhui CDC.

### RT-PCR and nested PCR amplification

A partial *pol* fragment was amplified and sequenced with an in-house protocol, as previously described [16,17]. Briefly, viral RNA was extracted from plasma using a QIAamp Viral RNA Mini kit (Qiagen, Valencia, CA, USA). HIV-1 cDNA was obtained and amplified using a one step RT-PCR kit (TaKaRa Biotechnology Co, Dalian, China) with primers RT21 (5’-CTG TAT TTC TGC TAT TAA GTC TTT TGA TGG G-3’, 3509–3539 nt, HBX2) and MAW26 (5’-TTG GAA ATG TGG AAA GGA AGG AC-3’, 2027–2050 nt, HBX2). The RT-PCR product was then used as a template and the nested PCR primers PRO-1 (5’-CAG AGC CAA CAG CCC CAC CA-3’, 2147–2166 nt, HBX2) and RT4R (5’-CTT CTG TAT ATC ATT GAC AGT CCA GCT-3’, 3299–3327 nt, HBX2) were used to amplify a *pol* fragment that included the whole protease (PR) and partial reverse transcriptase (1–250 amino acids, RT). The PCR products were purified by a gel extraction kit (Qiagen, Valencia, CA) and bidirectly sequenced using BigDye terminator sequencing technology (Applied Biosystems, Foster City, CA, USA) on an Applied Biosystems 3730 Sequencer [18,19].

### HIV-1 subtyping

The sequences of the *pol* fragment were aligned with reference sequences from the Los Alamos database using the BioEdit 7.0 software. HIV-1 subtype was determined by phylogenetic analyses using the neighbor-joining method [20]. For specimens of unknown HIV-1 subtype, bootscanning analyses were performed to screen for a recombination breakpoint using Simplot 3.5 software.

### Drug resistance genotyping of HIV-1 isolates

The *pol* sequences were compared with consensus B sequence in the Stanford HIV Drug Resistance Database (HIVdb; http://hivdb.stanford.edu), and surveillance/transmitted HIV-1 drug resistance mutations (SDRMs) were identified according to the 2009 World Health Organization (WHO) list [21]. The levels of drug resistance were analyzed with the HIVdb algorithm.

### Statistical analysis

Median (standard deviation, SD), range and frequencies (%) were used to describe patients’ characteristics. The frequency of SDRMs was also calculated. Statistical analyses were performed with SPSS version 17.0 (SPSS Inc., Chicago, IL, USA).

## Results

### Characteristics of participants

The median age of the 139 newly diagnosed HIV-1-infected MSM was 31.6 years (range 18–68), and half (48.2%) of them were youths (between 18–29 years old) (Table 1). A majority of subjects were of Han ethnicity, 65.5% were single, and 20.1% were married. Most participants (93.6%) had completed middle school or higher education. The main occupations were business service personnel (28.1%), officer/worker (19.4%), jobseeker/housekeeper (15.1%), and farmer (13.6%). A quarter of the subjects had sexually transmitted diseases in addition to HIV, and 80.8% of the individuals had CD4 counts equal to or above 350 cells/μl (Table 1).

**Table 1:** Characteristics of 139 HIV-1-infected treatment-naive MSM

**Characteristic**	**Cases (n)**	**Proportion (%)**
Age (years)		
18–29	67	48.2
30–39	42	30.2
40–49	22	15.8
50+	6	4.3
Ethnicity		
Han	136	97.8
Other	3	2.2
Marital status		
Divorced	15	10.8
Single	91	65.5
Married and having spouse	28	20.1
Unknown	5	3.6
Highest education achieved		
Elementary school	9	6.4
Middle school	45	32.4
High school	37	26.6
College and above	48	34.5
Occupation		
Business service personnel	39	28.1
Officer/worker	27	19.4
Jobseeker/housekeeper	21	15.1
Farmer	19	13.6
Student	9	6.5
Teacher/doctor	6	4.3
Other	18	12.9
Sex transmitted diseases		
Yes	39	28.1
No	81	58.3
Unknown	19	13.6
CD4 count: median (SD), range	446 (213), 49–1182	
<200	8	6.4
200–349	16	12.8
350–499	56	44.8
≥500	45	36.0

### HIV-1 subtypes and recombinant strains

A total of 133 (95.7%) specimens were successfully amplified and sequenced. According to a phylogenetic tree based on a 1.3 kb *pol* fragment (2147–3462 nt, HBX2) using the neighbor-joining method, two circulating recombinant forms (CRF) and one subtype were identified. The CRF01_AE strain was identified in 74 (55.6%) persons, CRF07_BC in 43 (32.3%) individuals, and subtype B in seven (5.3%) subjects (Table 2). Nine unique recombinant forms (URF) were determined according to phylogenetic inference and bootscanning analyses, including six CRF01_AE/B, two CRF01_AE/CRF07_BC, and one CRF01_AE/CRF01_B/CRF07_BC recombinant strain [20]. In the 35 specimens obtained from six northern cities (Fuyang, Bozhou, Huaibei, Huainan, Suzhou and Bengbu), CRF01_AE was found to be the predominant subtype (74.3%, 26/35). The dominance of CRF01_AE was also observed among MSM residing in the cities along the Yangtze River, such as Chizhou (100%, 1/1), Ma’anshan (66.7%, 4/6), Wuhu (58.8%, 10/17) and Tongling (50%, 2/4). It was also found in the central cities of Hefei (52.9%, 18/34) and Liu’an (50%, 1/2). In contrast to these high percentages of CRF01_AE, CRF07_BC was found to constitute the majority of infections in Bozhou (75%, 3/4), a northern city, and Anqing (64.3%, 9/14), a central city.

**Table 2:** Distribution of HIV-1 subtypes among newly diagnosed MSM (n = 133)

**Region**	**City**	**Cases (n)**	**HIV-1 subtypes (n)**			
			**CRF01_AE**	**CRF07_BC**	**B**	**Other**
Northern	Fuyang	5	3	0	1	1
	Bozhou	4	1	3	0	0
	Huaibei	2	2	0	0	0
	Huainan	12	10	1	1	0
	Suzhou	3	3	0	0	0
	Bengbu	9	7	0	0	2
Central	Hefei	34	18	13	1	2
	Liu’an	2	1	1	0	0
	Anqing	14	4	9	1	0
Southern	Ma’anshan	6	4	2	0	0
	Wuhu	17	10	3	1	3
	Tongling	4	2	2	0	0
	Chizhou	1	1	0	0	0
	Xuancheng	13	5	6	1	1
	Huangshan	7	3	3	1	0
Total (%)		133 (100)	74 (55.6)	43 (32.3)	7 (5.3)	9 (6.8)

### Identification and interpretation of surveillance/transmitted drug resistance mutations

Four cases were found have SDMRs: two from Anqing, one from Ma’anshan and one from Xuancheng. The rate of transmitted drug resistance (TDR) was 3.0% (4/133) for all ART drug classes, including nucleoside reverse transcriptase inhibitor (NRTIs)-related resistance in 1.5% (2/133) of the individuals, non-nucleoside reverse transcriptase inhibitor (NNRTIs)-related in 1.5% (2/133) of the subjects, and no PI-related resistance. The sole NRTI-related SDRM was L210W, detected in two individuals infected with CRF01_AE strains (from Anqing and Ma’anshan). The two NNRTI-related SDRMs were Y181C and G190A, found in persons infected with CRF01_AE (from Xuancheng) and B (from Anqing) strains, respectively (Table 3). L210W confers a low level of resistance to zidovudine (AZT) and stavudine (D4T). Y181C confers a high level of resistance to nevirapine (NVP) and an intermediate level of resistance to efavirenz (EFV), etravirine (ETR) and rilpivirine (RPV). G190A confers a high level of resistance to NVP and an intermediate level of resistance to EFV. However, two other mutations which confer resistance to RTIs, A98G and V79D, were not included in the 2009 WHO SDRM list but were identified here. A98G confers a low level of resistance to EFV and NVP, and while V179D in a URF strain confers a low level of resistance against EFV and an intermediate level of resistance against NVP, V179D in CRF01_AE does not affect resistance to any RT drugs. T69A/N/S, V179E and P236L were polymorphism mutations against RTIs.

**Table 3:** Frequency of surveillance/transmitted drug resistance mutations

**Drug class**	**Mutant sites**	**Number**	**Prevalence (%)**
NRTI	L210W	2	1.50
NNRTI			
	Y181C	1	0.75
	G190A	1	0.75
Total		4	3.00

Genotyping analysis revealed that none of the specimens had SDRMs against PIs. However, some accessory mutations that did not affect susceptibility to ARV drugs were found in the protease gene, including L10I/L/V (3.0%; 4/133), V11I/V (3.0%; 4/133), L33F (2.3%; 3/133), and A71A/V/T (12.8%; 17/133).

## Discussion

Our findings showed that CRF01_AE accounted for the majority (55.6%) of HIV-1-infections in MSM of Anhui province. This is consistent with the results of a national survey of HIV-1 molecular epidemiology in 2006, which reported that CRF07_BC (35.5%), CRF01_AE (27.6%), CRF08_BC (20.1%), and subtype B' (9.6%) were the four predominant HIV-1 strains in China [22]. Furthermore, CRF01_AE has replaced subtype B as the most common subtype among MSM [16,22]. The dominance of CRF01_AE was observed among MSM in coastal cities and provinces, such as Tianjin (88.9%), Jiangsu (70%), Shandong (77.8%), and Zhejiang (100%), in one province of central China, Shanxi (84.6%), and in one province of western China, Gansu (50%) [23].

In this study, a high proportion of MSM (30.9%) were married or divorced, raising concerns about the risk of acquiring HIV-1 from or transmitting HIV-1 to their sexual partners. Various forms of HIV transmission, including both homosexual and heterosexual behavior, likely exist among MSM of Liaoning province [24]. Therefore, further study should be carried out to determine which subtypes of HIV-1 predominantly infect the heterosexuals and IDUs.

We focused on HIV-1-infected treatment-naive MSM in Anhui province, and found that the overall TDR rate was 3.0%, slightly lower than the national level and Liaoning province [23,24]. The first nationwide survey of TDR in HIV-1-infected treatment-naive individuals was conducted in 2005, and the national TDR rate was reported to be 3.8% [4]. In 2011, the overall TDR rate was 4.9% among HIV-1-infected treatment-naive MSM in 19 Chinese provinces/cities (Anhui province was not included) [23]. According to an investigation in Liaoning province, 4.5% of ART-naive MSM had drug-resistant strains [24].

We report that 1.5% of samples were resistant to NRTIs, 1.5% of samples were resistant to NNRTIs, and none were resistant to PIs. Specific mutations included L210W, Y181C, and G190A, which confers different levels of drugs resistance. However, we detected many minor mutations which could compensate for the reduced fitness of resistant mutants, even though the minor mutations have not previously been associated with decreased in vitro susceptibility. Other commonly observed mutations were T69A/N/S, V179E, L10I/L/V, V11I/V, L33F, and A71A/T/V; none of these mutations is known to confer drug resistance to NRTIs, NNRTIs, or PIs.

Specific polymorphisms may occur in particular subtypes. For example, L10V, V11I/V, L33F, T69A/N/S, and P236L were present only in individuals with CRF01_AE strains. These mutations do not affect the susceptibility to ARV drugs. However, they might influence the emergence of drug-resistant viruses by modifying drug susceptibility and/or virus replication [25]. The present study found that substitutions of A71T/V and L10I often appeared in CRF07_BC, which was different from other studies reporting that substitutions of A71T/V often appeared in B viruses, whereas L10I was frequently discovered in CRF01_AE viruses [26]. A98G and V179E were excluded from the WHO list of SDRMs because of high occurrence in viruses from treatment-naive individuals. A98G has been shown to confer low-level resistance against EFV and NVP, and V179D in CRF01_AE/CRF07_BC recombinant viruses has been shown to confer low-level resistance against EFV and intermediate level resistance against NVP, while V179D in CRF01_AE viruses does not affect resistance to any RT drugs (data not published). This phenomenon is worthy of further study.

Our data represent only TDR in HIV-1-infected treatment-naive MSM in Anhui province; therefore, the results may not be representative of other areas. Nevertheless, these data warn us that HIV transmitted-drug-resistant isolates may be prevalent among HIV-1-infected treatment-naive MSM, which will no doubt compromise the success of future ART in these patients.

There were several limitations in our study. First, although all the subjects were newly diagnosed as HIV-infected, the date of their infections was not known. However, it is suggested that a substantial proportion of the subjects were infected recently, because more than half of the individuals had CD4 counts of ≥350 cells/μl, and almost half were between 18–29 years old. Second, the prevalence of TDR might be underestimated, because some mutations may have disappeared over time due to lack of replicative fitness. However, some NNRTI-related mutations, such as K103N, can persist for a long period of time [27,28].

## Conclusions

This study is the first to describe HIV diversity and TDR prevalence in ART-naive, HIV-1-infected MSM in Anhui province. CRF01_AE was found to be a major subtype, TDR prevalence was relatively low, and most TDR cases were associated with RTIs. These findings provide baseline information of HIV-1 transmitted-drug-resistant mutations, which has implications for HIV/AIDS prevention and treatment programs among MSM patients in China. Further studies of TDR are necessary to determine the trend of TDR in Anhui province and to optimize treatment regimens in the future.

## Competing interests

The authors declare that they have no conflicts of interest.

## Authors’ contributions

YS and BS designed and conducted the study and prepared and edited the manuscript. JW carried out the molecular genetic studies and drafted the manuscript. YQ and LM participated in the molecular genetic studies and prepared and drafted the manuscript. LJ, AL and XC participated in data interpretation and performed the statistical analysis. All authors read and approved the final manuscript.
